# AI-Driven Quantitative Dental Imaging: A Clinical Framework for Assessing Root Resorption Across Treatment Modalities

**DOI:** 10.3390/dj14070392

**Published:** 2026-06-25

**Authors:** Atanaz Darvizeh, Saman Fouladi, José Antonio González Sánchez, Guillermo Doria Jaureguizar, Oriol Quevedo, Fernando de la Iglesia Beyme, Funda Goker, Massimo Del Fabbro

**Affiliations:** 1 Department of Dentistry, UIC Barcelona International University of Catalonia, 08195 Barcelona, Spain; atanazdarvizeh@uic.es (A.D.); jagonzalez@uic.es (J.A.G.S.); guille_doria@uic.es (G.D.J.); uriquevedo@uic.es (O.Q.); fdelaiglesia20@gmail.com (F.d.l.I.B.); 2 Department of Computer Science, Università degli Studi di Milano, 20133 Milan, Italy; 3 Department of Oral and Maxillofacial Surgery, Faculty of Dentistry, Istanbul Aydın University, 34295 Istanbul, Turkey; funda.goker@unimi.it; 4 Fondazione IRCCS Ca’ Granda Ospedale Maggiore Policlinico, 20122 Milan, Italy; 5 Department of Biomedical, Surgical and Dental Sciences, University of Milan, 20122 Milan, Italy

**Keywords:** artificial intelligence, dental imaging, orthodontic treatment modalities, clear aligners, fixed appliances, root resorption

## Abstract

**Background:** Orthodontically induced external root resorption (ERR) may affect long-term tooth stability, requiring a reliable assessment of root length changes. This study developed an artificial intelligence (AI)-based framework for automatic segmentation and quantitative evaluation of root resorption, comparing fixed appliances and clear aligner therapy. **Methods:** A dataset of 100 anonymised orthopantomographic (OPG) radiographs (50 fixed appliance, 50 clear aligner) obtained before and after orthodontic treatment was analysed. A U-Net model was trained for automatic tooth segmentation and quantitative assessment of tooth length changes. Measurements were computed from both AI-predicted and Dentist-annotated masks using pixel-based Python analysis (Python version 3.6), and pre-post differences were compared between methods. **Results:** The segmentation model achieved high performance with Intersection over Union values up to 89% and Dice Similarity Coefficients up to 95%. Quantitative analysis demonstrated significant reductions in root length following orthodontic treatment in both modalities (*p* < 0.0001). In the fixed appliance group, AI-based measurements showed an average root length reduction of 8.95%, while human measurements indicated a reduction of 10.16%. In the clear aligner group, AI measurements demonstrated a reduction of 2.81%, compared with 4.98% in human measurements. Root resorption was significantly greater in the fixed appliance group than in the clear aligner group (*p* < 0.0001). AI-derived measurements showed strong agreement with expert evaluations. **Conclusions:** AI-based analysis of panoramic radiographs may provide a reliable and reproducible approach for quantifying orthodontically induced root resorption. The findings suggest that clear aligner therapy was associated with lower root length reduction; however, larger multi-centre studies and external validation are required before clinical implementation.

## 1. Introduction

Orthodontically induced external root resorption (ERR) is a common adverse effect of orthodontic treatment and remains a major clinical concern due to its irreversible nature and potential impact on long-term tooth prognosis [1,2]. Accurate detection and assessment of root resorption are therefore critical for risk stratification, treatment plan, and clinical decision-making [3].

In routine clinical practice, the diagnosis of root resorption is largely based on the visual interpretation of two-dimensional radiographs or cone-beam computed tomography (CBCT) by orthodontists or dentomaxillofacial radiologists. This approach, however, is time-intensive and inevitably influenced by observer experience and image quality, particularly when early or subtle resorptive changes are involved [4].

In recent years, artificial intelligence, in particular deep learning, has increasingly been adopted in dental and maxillofacial radiology to support automated image analysis. Growing evidence indicates that convolutional neural networks and related models are capable of identifying orthodontically induced root resorption on CBCT images with high accuracy [5,6,7,8,9,10]. Nevertheless, a robust model is particularly required for 2D radiographic images, which are widely used in clinical practice but are subject to geometric distortion, variable magnification, and anatomical superimposition that can affect measurement accuracy [11]. These models are capable of learning complex spatial patterns associated with apical and lateral root surface changes that may be difficult to consistently identify by clinician assessment. Importantly, AI-based systems offer the advantages of reproducibility, scalability, and objective quantitative assessment [7].

Beyond binary detection, more advanced deep learning frameworks have been developed to enable two-dimensional segmentation and quantitative assessment of root resorption severity. Automated volumetric and linear measurements obtained from radiographic images have demonstrated close agreement with expert evaluations, indicating that AI may support not only diagnostic classification but also accurate and reproducible quantification of treatment-related outcomes [6]. Such capabilities are particularly relevant in orthodontics, where treatment-induced changes occur gradually and may require longitudinal monitoring.

Comparative studies evaluating AI performance against experienced human experts consistently report non-inferior, and in some cases superior, diagnostic accuracy for AI-based models [5,6,7]. These findings highlight the potential role of AI as a clinical decision-support tool in orthodontics, with the potential to reduce diagnostic variability and streamline image interpretation. Furthermore, hierarchical and feature-enhanced deep learning strategies have been introduced to improve performance in diagnostically demanding situations, including cases of canine-induced or localized root resorption [8].

Despite the expanding literature on AI-based detection and quantification of root resorption [5,6,7,8,9], most published studies have focused primarily on algorithm development and technical validation rather than clinically oriented comparisons between different orthodontic treatment modalities. To the best of our knowledge, no studies have used artificial intelligence to directly compare the severity of orthodontically induced root resorption between fixed appliances and clear aligner therapy, nor to perform a direct comparison between AI-based assessments and human expert evaluations within the same study design.

The present study aimed to develop an AI-based framework for quantitatively assessing and comparing orthodontically induced root resorption associated with fixed appliances and clear aligner therapy.

## 2. Materials and Methods

The study was conducted in accordance with the Declaration of Helsinki and approved by the Ethics Committee (CEIM) of the International University of Catalonia (reference number: END-ECL-2023-02; approval date: 8 November 2023). To achieve this objective, a dataset comprising pre- and post-treatment Orthopantomographic images from patients treated with both modalities was collected, with analysis focused on four selected teeth, specifically mandibular incisors. The analysis focused on mandibular incisors primarily due to their more favourable imaging characteristics on panoramic radiographs. In this region, reduced anatomical superimposition, particularly from adjacent structures such as the maxillary sinus and other overlapping anatomical contours, allows for clearer visualization of the full tooth profile. This facilitates more consistent and reliable segmentation by both clinicians and the AI model. From a methodological perspective, selecting teeth with more clearly delineated boundaries was essential to ensure high-quality ground truth annotations and to minimize ambiguity during model training. This approach was intended to enhance segmentation accuracy and improve the robustness of quantitative measurements derived from both AI-based and expert-based masks.

A U-Net deep neural network was trained to perform automatic tooth segmentation. The trained model was applied to segment the entire length of four selected teeth in both pre- and post-treatment images for each orthodontic modality. Root length was then calculated for each tooth, with the measurement of tooth length performed along the major tooth axis, and the difference in tooth length before and after orthodontic treatment was computed on the test dataset. Because orthodontically induced external root resorption is reflected by a reduction in root length, pre- and post-treatment tooth length measurements were used to quantitatively estimate resorption severity.

Subsequently, the extent of root length reduction was compared between fixed appliances and clear aligner therapy to determine which orthodontic approach was associated with greater root resorption. Additionally, expert-annotated segmentations provided by experienced dentists were utilized to calculate root length changes for the same test cases. The differences between AI-based measurements and expert evaluations were analyzed to assess the agreement and reliability of the proposed model.

### 2.1. Dataset

The dataset consisted of anonymized OPG radiographs collected from patients who underwent orthodontic treatment with either fixed appliances or clear aligner therapy. A total of 200 panoramic radiographs were initially screened, including 100 images from patients treated with fixed appliances and 100 images from patients treated with clear aligners. Inclusion criteria were as follows: patients who had undergone orthodontic treatment with either fixed appliances or clear aligners; availability of pre- and post-treatment panoramic radiographs; clearly visible mandibular incisors and images of sufficient quality to allow reliable segmentation and tooth-length measurement. Exclusion criteria included poor image quality, severe distortion or positioning errors, marked anatomical superimposition affecting the mandibular incisor region, previous endodontic treatment of the selected teeth, extensive restorations or prosthetic crowns involving the selected teeth, missing or severely malformed mandibular incisors, history of dental trauma, periapical or periodontal pathology affecting root morphology, and systemic or local conditions potentially associated with abnormal root resorption.

After applying the predefined inclusion and exclusion criteria and image-quality requirements, 50 radiographs per group were included in the final analysis. The overall patient age range was 13–45 years across the full study cohort. All patients underwent orthodontic treatment between January 2019 and December 2023. All OPG radiographs were obtained from a single clinical centre using the routine standardized panoramic radiographic protocol of the institution. Image acquisition was performed under the supervision of the treating expert orthodontist, and the same clinical imaging workflow was applied for all included patients. No radiographs from external institutions or multiple centres were included.

All selected images were anonymized prior to analysis to ensure patient privacy. Image selection and initial verification were independently performed by two blinded clinicians (A.D. and J.G.), both with more than 10 years of experience in dental radiographic evaluation. Inter-observer agreement for image selection was excellent, with an overall agreement rate of 92% and a Cohen’s kappa coefficient of κ = 0.84. Discrepancies were resolved by consensus before final dataset inclusion, and the final reference annotations were used for analysis and validation of the AI-based segmentation results.

The imaging dataset analysed in the present study is independent and does not overlap with any previous publications by the authors. Specifically, the previously published systematic review and meta-analysis synthesized data from existing published studies and did not include any original patient-level OPG data from the present AI-based analysis.

### 2.2. Study Workflow

To ensure a robust and objective comparison, statistical analysis was performed to compare human-based vs. AI-based root length measurements and root resorption in both treatment groups (fixed appliances vs. CA) with paired non-parametric tests (Wilcoxon test). We also compared fixed appliance vs. Invisalign data using unpaired non-parametric tests (Mann–Whitney U test) in both Human-based and AI-based settings. Both tooth-based and patient-based analyses were performed. Non-parametric tests were used because the data distributions were not Gaussian, as evaluated by the D’Agostino and Pearson omnibus normality test. Statistical analysis was performed using GraphPad Prism Version 5 (GraphPad Software, Boston, MA, USA). A *p*-value of 0.05 was considered the significance threshold.

### 2.3. Methods

**Figure 1:**
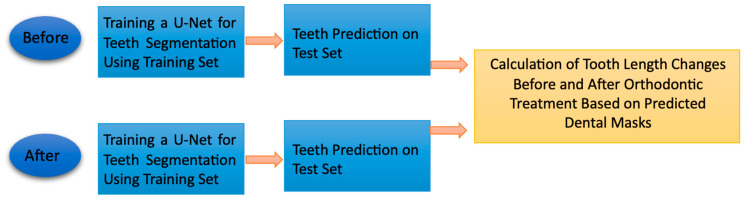
Overview of the U-Net–based workflow used to compute tooth length changes before and after fixed or clear aligner orthodontics.

Figure 1 illustrates the workflow of the proposed method for quantitative tooth length analysis. Dental images acquired before and after orthodontic treatment were processed separately. In both cases, a U-Net deep learning model trained on the training dataset was employed to perform automatic tooth segmentation on the test dataset.

The predicted segmentation masks obtained from pre-orthodontic and post-orthodontic images were used to calculate tooth length for each selected tooth. Tooth length changes were then computed by subtracting the post-treatment length from the pre-treatment length, providing a quantitative measure of orthodontically induced root resorption. Absolute measurements on panoramic radiographs are inherently influenced by magnification and geometric distortion. To address this limitation, a proportional (%) approach was adopted, allowing for consistent intra-subject comparison between pre- and post-treatment images. Panoramic radiographs are inherently affected by geometric distortion, magnification variability, projection-related differences, and anatomical superimposition, which may limit the precision of absolute root length measurements. To reduce measurement variability, the assessors were calibrated before the final evaluations, and all human- and AI-derived measurements were performed on the same radiographic images using a standardized Python-based measurement workflow. Since both human and AI measurements were obtained from the same pre- and post-treatment radiographs, any distortion related to panoramic imaging was expected to affect both measurement approaches similarly and was therefore not expected to introduce systematic bias in the comparison between AI-derived and expert-derived measurements. However, such distortion may still limit the precision of absolute measurements compared with true anatomical values. For this reason, tooth lengths were first measured in pixel units, and root length reduction was then expressed as a proportional (%) change relative to the corresponding pre-treatment length, calculated as follows:

$$Percentage\;root\;length\;reduction\left(\%\right)=\frac{\left(pr\mathrm{e-}treatment\;tooth\;length\right)-\left(pos\mathrm{t-}tratment\;tooth\;lenght\right)}{\left(pr\mathrm{e-}treatment\;tooth\;length\right)}\times 100$$

This method has been widely used in similar contexts, as relative changes are less affected by imaging variability than absolute linear measurements. This procedure was applied independently to both orthodontic treatment modalities, namely fixed appliances and clear aligner therapy.

In the subsequent step, the magnitude of tooth length reduction was compared between the two orthodontic modalities to determine which treatment approach was associated with greater root resorption. The U-Net model was trained exclusively on the training dataset, while all segmentation, measurement, and comparative analyses were performed on the independent test dataset.

In addition to the AI-based analysis, expert-derived masks (ground truth) were generated by two experienced clinicians (A.D. and J.G.) using 3D Slicer software (version 5.6; 3D Slicer Community, Boston, MA, USA) to segment teeth in the same test images. 3D Slicer is a widely adopted open-source platform for medical image analysis and segmentation, and has been extensively validated in the literature [12]. Segmentations were performed independently and subsequently reviewed to reach consensus, ensuring consistency across annotations.

Tooth lengths were calculated from these expert-derived masks, with measurements performed along the major tooth axis, both before and after orthodontic treatment for each modality.

To ensure consistency, reproducibility, and to minimize potential sources of error or observer bias, all measurements were performed using a custom-developed Python pipeline, which standardized the computation process across all cases and conditions. The differences in tooth length obtained from expert segmentations were then analysed in parallel with those derived from the U-Net–predicted masks.

Finally, comparisons were conducted (i) between fixed appliances and clear aligner therapy with respect to tooth length reduction, and (ii) between AI-based measurements and expert-based measurements, enabling an assessment of both treatment-related differences in root resorption and the agreement between automated and human expert evaluations.

### 2.4. Neural Network Architecture

U-Net is a fully convolutional neural network specifically designed for biomedical image segmentation [13]. It follows an encoder–decoder architecture in which the encoder progressively captures high-level semantic features through successive convolution and down-sampling operations, while the decoder restores spatial resolution via up-sampling layers. A key characteristic of U-Net is the use of skip connections between corresponding encoder and decoder stages, which allow the network to combine fine-grained spatial information with deep semantic representations. This design enables precise localization and has proven particularly effective for segmenting anatomical structures in medical images, even when training data are limited [14,15].

**Figure 2:**
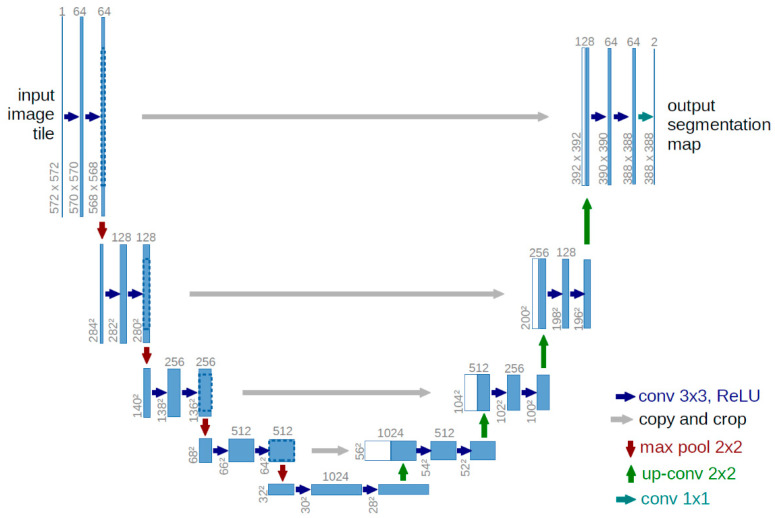
The Architecture of the Standard U-Net Neural Network.

Figure 2 presents the architecture of the standard U-Net, including its principal components and network layers.

### 2.5. Implementation Details

In our implementation, the network input is a single-channel image of size 160 × 160, and the output is a corresponding segmentation mask with the same spatial resolution. A total of 39 images were used for training and 11 images for testing. The encoder consists of four down-sampling blocks, each containing two 3 × 3 convolutional layers with ReLU activations followed by 2 × 2 max pooling, doubling the number of feature channels at each step (from 64 up to 512). At the bottleneck, two convolutional layers with 1024 filters extract deep semantic features. The decoder mirrors the encoder: each upsampling stage uses 2 × 2 transposed convolutions to increase spatial resolution, followed by concatenation with the corresponding encoder feature maps (via skip connections) and two 3 × 3 convolutional layers with ReLU activations. A final 1 × 1 convolution with a sigmoid activation produces the binary segmentation output [16].

This architecture was trained end-to-end using 5-fold cross-validation to enhance generalization and mitigate overfitting on limited data. During training, data augmentation was applied to improve robustness and reduce overfitting, including small-angle rotations, slight zooming, horizontal flipping, and the addition of very light noise, which are commonly considered safe and effective transformations for medical imaging tasks [17,18].

The networks were trained for a maximum of 200 epochs using the Adam optimizer (learning rate = 1 × 10^−4^). To further prevent overfitting and reduce unnecessary training, an early stopping strategy was employed, monitoring the validation loss and terminating training when no improvement was observed for a predefined number of epochs. The model was compiled using a compound loss function that combines binary cross-entropy and Dice loss, enabling a balance between pixel-wise classification accuracy and overlap-based similarity.

Model performance was evaluated using the Dice Similarity Coefficient (DSC) and Intersection over Union (IoU), defined as:

$$DSC=\frac{2|A\cap B|}{|A|+|B|}$$

$$IoU=\frac{|A\cap B|}{|A\cup B|}$$

Both the Dice coefficient and Intersection over Union serve as key evaluation metrics, quantifying the degree of overlap between predictions and true labels, with higher values indicating better segmentation quality [19].

The image processing and deep learning methodologies in this study were implemented in a Python environment (version 3.6; Python Software Foundation, Wilmington, DE, USA) using the Keras library and TensorFlow framework (Google LLC, Mountain View, CA, USA). All experiments were performed on Google Colaboratory (Colab; Google LLC, Mountain View, CA, USA), a cloud-based platform that enables execution of Python code directly in the browser without the need for local computational resources. The Colab environment provided access to a GPU (1× Tesla K80 with 12 GB VRAM, GDDR5 memory, 2496 CUDA cores, and compute capability 3.7), a CPU with 1 core and 2 threads based on Intel Xeon processors operating at 2.3 GHz, approximately 12.6 GB of RAM, and around 33 GB of disk storage.

## 3. Results

### 3.1. Segmentation Results

Table 1 reports the segmentation performance of the U-Net model for tooth segmentation under two orthodontic conditions (fixed and clear aligner), evaluated before and after treatments using the IoU and DSC.

For the fixed appliance group, the model achieved an IoU of 87% and a DSC of 93% before treatment, and an IoU of 85% and a DSC of 92% after treatment. For the clear aligner group, the model achieved an IoU of 85% and a DSC of 91% before treatment, increasing to an IoU of 89% and a DSC of 95% after treatment. Overall, the U-Net model showed high segmentation performance across both orthodontic treatment groups and both timepoints.

Overall, the results demonstrate that the proposed U-Net model maintains robust segmentation performance across different orthodontic types and imaging conditions, with consistently high IoU and DSC values both before and after treatments.

**Figure 3:**
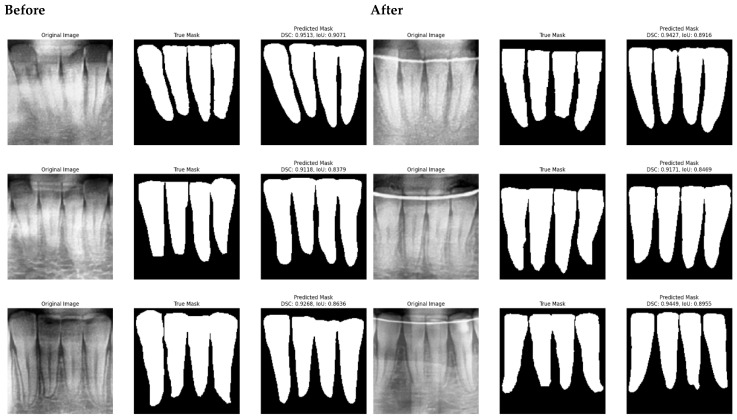
Representative Teeth Segmentation Results Before and After Fixed Orthodontic Treatment.

Figure 3 illustrates representative examples of teeth segmentation results on the test set for fixed type, showing images before and after orthodontic treatment. For each example, the first column shows the original teeth image, the second column presents the ground-truth mask annotated by an expert, and the third column displays the corresponding mask predicted by the proposed AI model. These images represent some samples from our test set.

**Figure 4:**
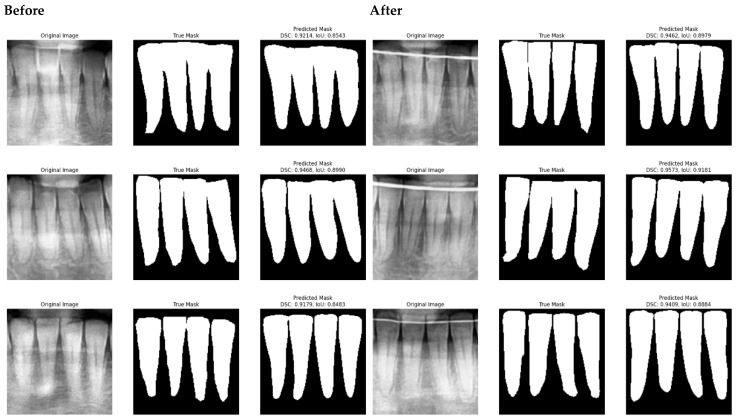
Representative Teeth Segmentation Results Before and After Clear Aligner Treatment.

Figure 4 illustrates representative examples of teeth segmentation results on the test set for the clear aligner type, showing images before and after orthodontic treatment. The columns correspond to the original teeth image, the expert-annotated ground-truth mask, and the mask predicted by the proposed AI model, respectively. Additional high-quality representative pre- and post-treatment tooth segmentation examples corresponding to Figure 3 and Figure 4 are presented in Appendix A.

After mask prediction by U-Net, the important part was to compare the lengths of each predicted tooth before and after treatment.

Tables S1 and S2 summarize the predicted lengths of the four teeth for each test image in fixed orthodontic and clear aligner, respectively.

In each table, we report both the AI-predicted masks by the U-Net model and the corresponding ground-truth masks manually generated by a human expert for the test set, allowing direct comparison between automated and reference measurements.

For every test image, separate rows are provided for each tooth. The reported values include the length (height) before treatment, the length after treatment, and the calculated difference (After − Before). All length measurements are expressed in pixel units.

Tables S1 and S2 provide a clear, detailed quantitative comparison of tooth-length changes associated with each orthodontic procedure, while also enabling evaluation of agreement between AI-based predictions and human-generated reference masks.

### 3.2. Quantitative Analysis

Following segmentation, quantitative tooth-length measurements were obtained from both AI-generated masks and expert-generated masks in the test dataset. Root length differences between pre-treatment and post-treatment images were calculated to estimate orthodontically induced root resorption. The statistical results obtained from the quantitative measurements are summarized below.

#### 3.2.1. Fixed Orthodontic Appliances

In the tooth-based analysis (*n* = 44 teeth), AI-based measurements showed a mean root length reduction from 139.18 ± 13.20 pixels before treatment to 126.73 ± 15.57 pixels after treatment, corresponding to an average decrease of 12.45 pixels (8.95%) (Table S3). Human-based measurements showed a similar pattern, with mean root length decreasing from 142.95 ± 14.36 pixels to 128.43 ± 15.85 pixels, corresponding to an average reduction of 14.52 pixels (10.16%) (Table S3).

Both AI-based and human-based evaluations demonstrated highly significant reductions in root length between pre- and post-treatment measurements (*p* < 0.0001).

Comparison between AI and human measurements revealed no statistically significant differences for baseline measurements, post-treatment measurements, or calculated root length differences (*p* = 0.1011, *p* = 0.4759, and *p* = 0.381, respectively), indicating a strong agreement between the automated system and expert evaluation.

The patient-based analysis (*n* = 11 patients) confirmed the results (Table S5). AI-based measurements showed a mean reduction of 12.45 pixels, while human measurements indicated a mean reduction of 14.52 pixels, with both analyses demonstrating statistically significant pre- to post-treatment differences (*p* = 0.001). No significant differences were detected between AI and human measurements in this analysis (all *p* > 0.05).

#### 3.2.2. Clear Aligner Therapy

For clear aligner orthodontic treatment, the tooth-based analysis (*n* = 44 teeth) showed a smaller magnitude of root length reduction. AI-based measurements demonstrated a decrease from 142.20 ± 14.13 pixels before treatment to 138.20 ± 16.23 pixels after treatment, corresponding to an average reduction of 4.00 pixels (2.81%) (Table S4).

Human-based measurements indicated a reduction from 144.80 ± 17.33 pixels to 137.59 ± 19.04 pixels, corresponding to an average decrease of 7.20 pixels (4.98%).

Both AI-based and human-based analyses showed statistically significant differences between pre- and post-treatment measurements (*p* < 0.0001).

When comparing AI and human measurements, no significant differences were observed for baseline values or post-treatment values (*p* = 0.3642 and *p* = 0.6593, respectively). However, the estimated root length reduction was significantly greater in human-based measurements compared with AI-based measurements (*p* = 0.0133).

In the patient-based analysis (*n* = 11 patients), AI-based measurements demonstrated an average reduction of 4.00 pixels (Table S6), whereas human-based measurements showed an average reduction of 7.20 pixels. Both methods detected statistically significant pre- to post-treatment changes (*p* = 0.0038 for AI and *p* = 0.0233 for human evaluation). The difference between AI- and human-derived root length reductions approached significance (*p* = 0.0618).

#### 3.2.3. Comparison Between Fixed Appliances and Clear Aligners

Direct comparison between orthodontic treatment modalities revealed a significantly greater root length reduction in the fixed appliance group compared with the clear aligner group.

Based on AI-derived measurements, fixed appliances resulted in a mean reduction of 8.95%, whereas clear aligners produced an average reduction of 2.81% (*p* < 0.0001 in tooth-based analysis and *p* = 0.0025 in patient-based analysis).

Human-based measurements showed a similar trend, with average root length reductions of 10.16% for fixed appliances and 4.98% for clear aligners, demonstrating statistically significant differences between the two treatment modalities (*p* < 0.0001 at the tooth level and *p* = 0.0137 at the patient level).

Overall, these findings indicate that the AI-based framework was capable of detecting orthodontically induced root resorption and produced measurements largely consistent with expert human evaluations, while also identifying greater root resorption associated with fixed orthodontic appliances than with clear aligner therapy.

## 4. Discussion

Orthodontically induced external root resorption represents a well-recognized biological response associated with orthodontic tooth movement and has been widely documented as one of the most relevant adverse effects of orthodontic therapy. The pathophysiology of this phenomenon is related to the mechanical forces applied during orthodontic treatment, which induce localized stress within the periodontal ligament and surrounding alveolar bone. This process may activate inflammatory pathways and odontoclastic activity, leading to progressive loss of root structure, particularly at the apical region. Although mild root shortening is considered a relatively common and often clinically acceptable consequence of orthodontic treatment, excessive resorption may compromise long-term tooth stability and therefore requires careful monitoring during therapy [1,2,3].

The present study investigated the feasibility of using an artificial intelligence–based segmentation framework to quantify orthodontically induced root resorption and to compare the extent of root length reduction between two different orthodontic treatment modalities. The quantitative results demonstrated that both AI-derived and human-derived measurements consistently detected significant reductions in root length following orthodontic treatment. These findings confirm that measurable structural changes in root morphology occur during orthodontic therapy and that automated image analysis methods are capable of capturing these changes with a level of sensitivity comparable to expert evaluation. Such observations are consistent with the current understanding that orthodontic tooth movement can induce remodelling processes affecting both the surrounding bone and the dental root surface [1,3].

The selection of mandibular incisors in the present study was primarily driven by methodological considerations related to image quality and segmentation reliability. Compared with other dental regions, mandibular incisors on panoramic radiographs are less affected by anatomical superimposition, allowing for clearer delineation of tooth boundaries. This is particularly relevant in the context of AI-based segmentation, where well-defined anatomical contours are essential for both accurate model training and reliable quantitative analysis. By focusing on teeth with more consistent radiographic representation, the study aimed to reduce variability associated with image interpretation and to enhance the reproducibility of both AI-derived and expert-derived measurements.

A major finding of this study is the strong agreement observed between AI-based measurements and expert-derived measurements, particularly in the fixed orthodontic treatment group. No statistically significant differences were detected between the two evaluation methods for baseline measurements, post-treatment measurements or calculated root length reductions. This result suggests that automated segmentation and measurement pipelines may provide reliable quantitative estimates of root length comparable to those obtained through expert manual assessment. Previous investigations employing deep learning techniques for the detection of external root resorption have similarly reported that convolutional neural networks can achieve diagnostic performance comparable to experienced clinicians, supporting the integration of AI systems into dental image analysis workflows [5].

The slight variation in segmentation performance between treatment groups and timepoints may be related to differences in image appearance, boundary definition, and post-treatment anatomical presentation on panoramic radiographs. However, this interpretation should be considered exploratory, as the study was not specifically designed to determine the specific imaging factors responsible for these differences.

In the clear aligner group, the present study observed a small but measurable discrepancy between AI-based and human-based estimations of root length reduction, with human measurements indicating slightly greater root shortening. This difference may reflect variations in segmentation boundary definition, particularly near the root apex, where anatomical contours can be more difficult to delineate in two-dimensional panoramic images. In addition, the relatively small magnitude of resorption in this group may have contributed to this discrepancy, as subtle apical changes are inherently more challenging to detect on OPG images. Minor discrepancies between automated and manual measurements have also been reported in previous deep learning studies, where such differences were attributed to annotation variability, image resolution, and subjective interpretation of anatomical landmarks by human observers [7].

Another relevant observation of the present study is the significantly greater magnitude of root length reduction associated with fixed orthodontic appliances compared with clear aligner therapy. Both AI-based and human-based analyses consistently demonstrated greater root shortening in the fixed appliance group. These findings may be explained by differences in biomechanical force application between the two treatment modalities. Fixed appliances typically exert continuous forces through brackets and archwires, potentially generating sustained mechanical stress within the periodontal ligament [20]. In contrast, clear aligner systems apply more controlled and intermittent forces due to the removable nature of the appliance, which may result in reduced biological stress on the root surface during tooth movement [21]. Previous studies investigating orthodontically induced root resorption have suggested that both the magnitude and duration of orthodontic forces play a significant role in determining the severity of resorptive changes [1,2].

The present findings are consistent with previous comparative studies and recent reviews evaluating external apical root resorption across orthodontic treatment modalities. Fang et al. reported in a systematic review and meta-analysis that clear aligner therapy was associated with less root resorption than fixed appliance treatment [22]. Similarly, Li et al., using CBCT-based assessment, found a lower prevalence and severity of apical root resorption in patients treated with clear aligners compared with fixed appliances [23]. More recent evidence, including the systematic review and meta-analysis by Singh et al. and the umbrella review and meta-analysis by Selvaraj et al., further supports the overall trend that clear aligner therapy is associated with a lower degree of external apical root resorption than fixed appliance therapy [24,25]. In agreement with these studies and with our recent systematic review and meta-analysis [26], the present work demonstrated lower root length reduction in the clear aligner group than in the fixed appliance group. Importantly, the present study extends the existing literature by applying an AI-based quantitative segmentation framework to panoramic radiographs and by directly comparing automated measurements with expert-derived assessments within the same study design.

The present study demonstrated greater root length reduction in the fixed appliance group than in the clear aligner group. Using AI-derived measurements, the mean reduction was 8.95% for fixed appliances and 2.81% for clear aligners, corresponding to a between-group difference of 6.14%. Human-based measurements showed a similar pattern, with reductions of 10.16% and 4.98%, respectively, corresponding to a difference of 5.18%. Although these differences were statistically significant, their clinical relevance should be interpreted cautiously. Orthodontically induced external root resorption is a well-recognized adverse effect of orthodontic tooth movement, and its clinical impact depends not only on the amount of root shortening but also on the remaining periodontal support, tooth mobility, symptoms, treatment duration, and individual susceptibility [1,2,3]. Previous literature has reported that orthodontically induced external apical root resorption is usually less than 2.5 mm and is generally classified as mild to moderate with limited clinical significance, whereas severe resorption is commonly defined as root shortening exceeding 4 mm or more than one-third of the original root length [27]. Therefore, the magnitude of reduction observed in the present study is likely to fall within the mild-to-moderate range and may not necessarily compromise long-term tooth stability in healthy patients. Nevertheless, the greater reduction observed with fixed appliances may still be clinically meaningful in susceptible individuals, including patients with pre-existing short roots, previous root resorption, periodontal compromise, prolonged treatment duration, or cases requiring extensive tooth movement, particularly because both the magnitude and duration of orthodontic forces have been implicated in the severity of root resorption [1,20]. These findings are consistent with previous literature, including our recent systematic review and meta-analysis, suggesting that clear aligner therapy is generally associated with lower external apical root resorption compared with conventional fixed appliances [26].

Nevertheless, even modest differences may become clinically relevant in susceptible individuals or in cases of prolonged treatment duration, underscoring the importance of careful radiographic monitoring throughout orthodontic therapy.

Beyond the comparison of orthodontic modalities, the present study also highlights the broader potential of artificial intelligence for quantitative monitoring of treatment-related structural changes in dentistry. Automated image analysis offers several advantages over conventional manual evaluation, including improved reproducibility, reduced observer variability, and the ability to analyze large imaging datasets efficiently. Recent developments in deep learning have demonstrated that AI models can successfully detect and quantify root resorption in radiographic and CBCT datasets, supporting their potential role as clinical decision-support tools in orthodontics and dentomaxillofacial radiology [8,28]. Importantly, while CBCT provides high-resolution three-dimensional imaging, its routine use may be limited by factors such as higher radiation exposure, cost, and accessibility. In contrast, panoramic radiography remains widely available and is commonly used in daily clinical practice. In this context, the ability of the proposed AI-based framework to reliably quantify root resorption using two-dimensional OPG images represents a clinically relevant advantage, as it enables scalable and cost-effective monitoring of treatment-related changes without the need for advanced imaging modalities.

Despite these promising findings, several methodological considerations should be taken into account when interpreting the results of this study. First, the analysis was performed using two-dimensional panoramic radiographs, which are known to introduce geometric distortion, variable magnification, projection-related differences, and anatomical superimposition. These factors may influence the precision of linear measurements, particularly when evaluating small structural changes at the root apex [29]. To reduce this limitation, root length changes were analysed using proportional pre- and post-treatment measurements rather than relying solely on absolute pixel values. This approach allowed each patient to serve as their own reference and reduced the influence of magnification and geometric distortion. In addition, all AI-derived and expert-derived measurements were performed using the same standardized Python pipeline, ensuring consistent measurement procedures across treatment groups and evaluation methods. Nevertheless, these precautions cannot completely eliminate the intrinsic limitations of two-dimensional OPG imaging.

Second, the study dataset was relatively limited in size, which may restrict the generalizability of the trained model to broader patient populations or imaging conditions [30]. In addition, the model was developed and evaluated using data acquired from a single clinical centre, which may introduce potential bias related to imaging protocols and patient population characteristics. Although data augmentation, 5-fold cross-validation, and early stopping were used to reduce overfitting and improve model generalization, external validation on independent multi-centre datasets remains necessary before clinical translation to ensure generalizability and robustness across different clinical settings. Future investigations, including larger multi-centre datasets and three-dimensional imaging modalities such as CBCT, may provide additional insights into the clinical applicability of AI-based root resorption assessment [28].

Third, the quantitative analysis was restricted to the four mandibular incisors. Although this selection was methodologically justified by the clearer radiographic boundaries and reduced anatomical superimposition of this region on panoramic radiographs, it may limit the generalizability of the findings to other tooth regions. Future studies should include additional dental regions to evaluate whether the proposed AI-based framework maintains comparable segmentation and measurement performance across teeth with more complex morphology and greater anatomical overlap.

Finally, the reproducibility of the AI-based measurements was not explicitly evaluated through repeated runs or perturbation-based testing. However, the inference process of the trained U-Net model is deterministic, as predictions are generated using fixed learned weights without stochastic variation during testing. Future research may also explore advanced deep learning strategies, including multi-scale feature extraction, hierarchical learning architectures, hybrid segmentation-classification pipelines, and longitudinal AI models capable of tracking morphological changes across multiple treatment timepoints [5,7].

## 5. Conclusions

This study demonstrated that an artificial intelligence-based segmentation framework can reliably quantify orthodontically induced root resorption from panoramic radiographs and provide measurements comparable to expert human evaluation. The proposed U-Net-based approach successfully detected significant reductions in root length following orthodontic treatment and showed strong agreement with manual measurements performed by experienced clinicians.

Quantitative analysis further revealed that root length reduction was significantly greater in patients treated with fixed orthodontic appliances compared with those treated with clear aligner therapy, suggesting potential differences in biomechanical effects between these treatment modalities.

Overall, the findings indicate that AI-assisted image analysis may represent a promising preliminary approach for objective and reproducible assessment of treatment-related root changes. However, larger multi-centre studies and external validation are required before clinical implementation.

## Figures and Tables

**Table 1:** Comparison of IoU and DSC for teeth segmentation before and after treatments using U-Net.

		IoU	DSC
Fixed Orthodontics	Before	87%	93%
	After	85%	92%
Clear Orthodontics	Before	85%	91%
	After	89%	95%

**Figure A1:**
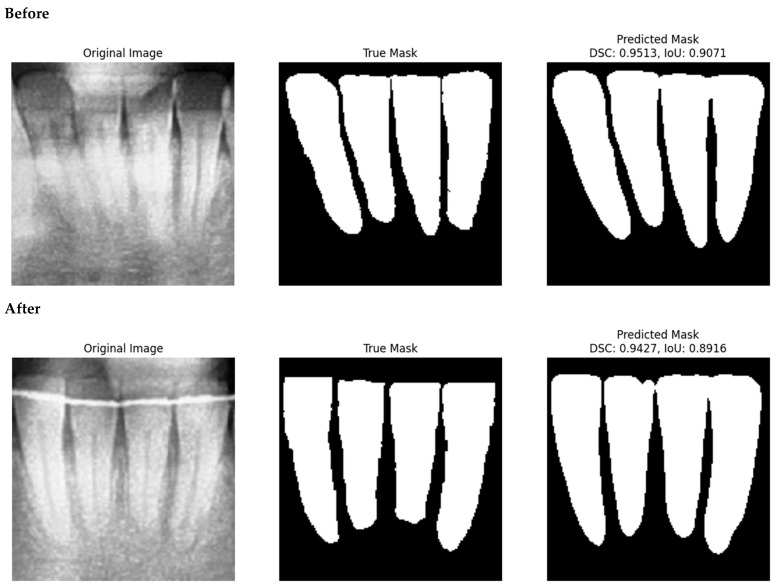
Fixed appliances: representative pre- and post-treatment segmentation example.

**Figure A2:**
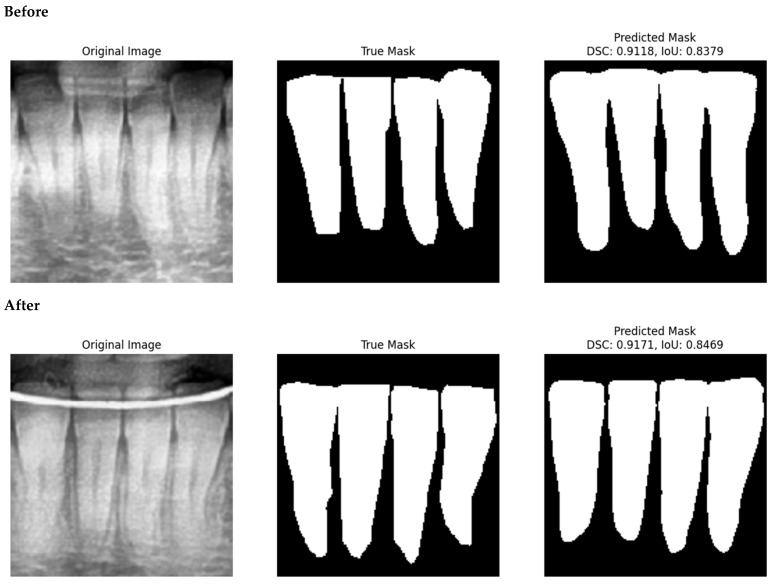
Fixed appliances: representative pre- and post-treatment segmentation example.

**Figure A3:**
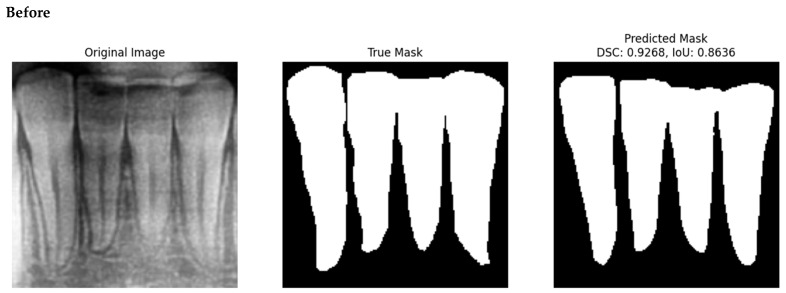

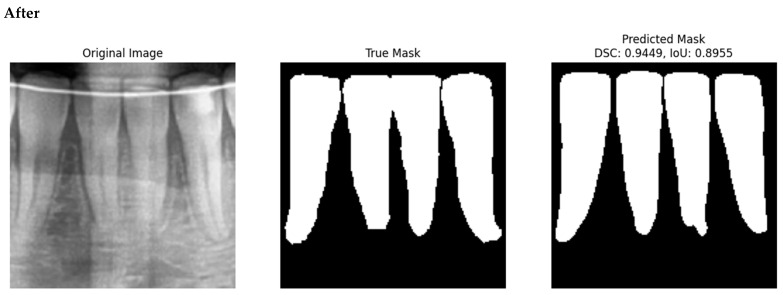
Fixed appliances: representative pre- and post-treatment segmentation example.

**Figure A4:**
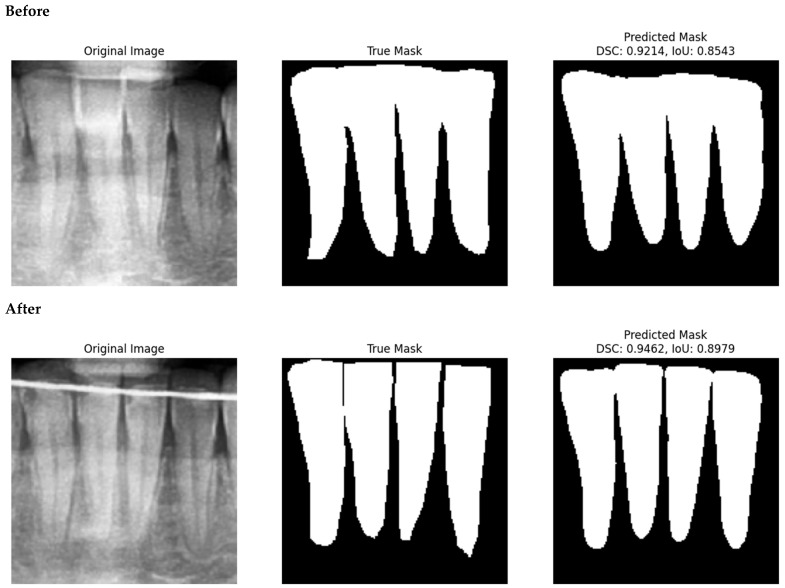
Clear aligners: representative pre- and post-treatment segmentation example.

**Figure A5:**
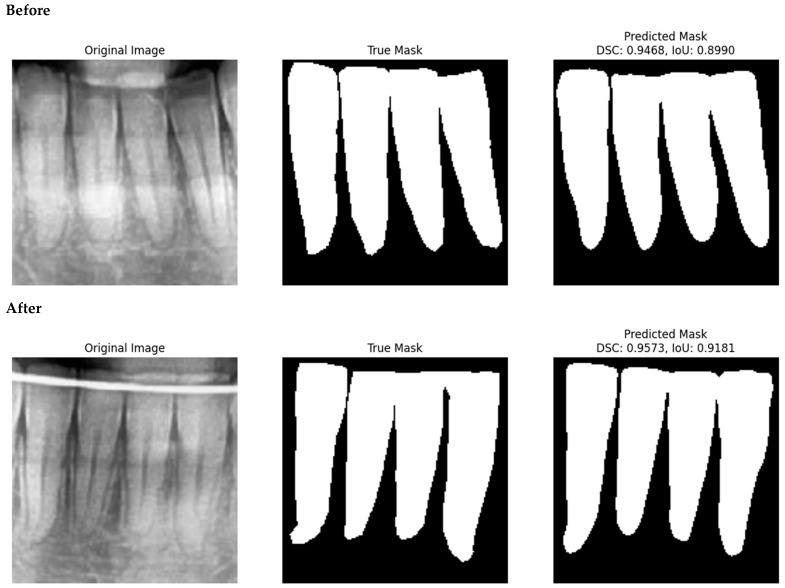
Clear aligners: representative pre- and post-treatment segmentation example.

**Figure A6:**
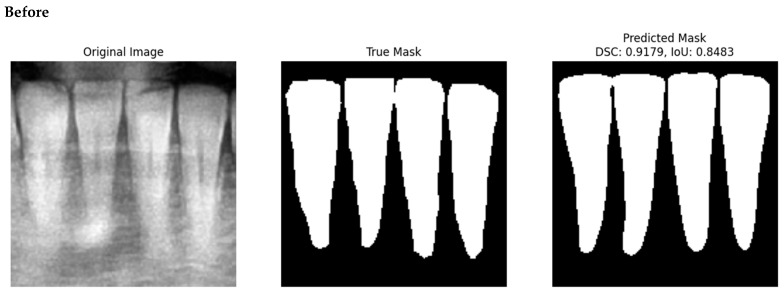

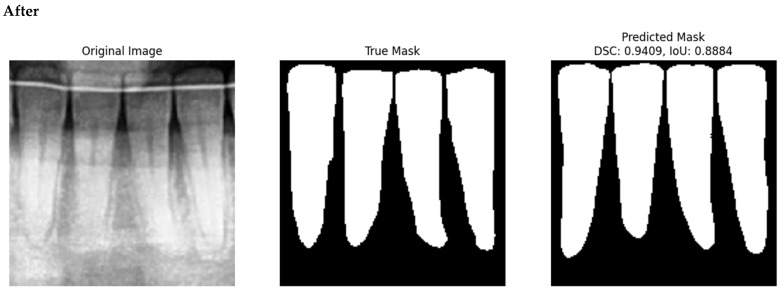
Clear aligners: representative pre- and post-treatment segmentation example.

## Data Availability

The data presented in this study are available on request.

## Supplementary Materials

The following supporting information can be downloaded at: https://www.mdpi.com/article/10.3390/dj14070392/s1, Table S1. Comparison of Tooth Length Measurements Before and After Fixed Orthodontic Treatment Based on U-Net Predicted Masks and Original Masks Provided by a Human. All data are expressed in pixels. Table S2. Comparison of Tooth Length Measurements Before and After Clear Aligner Treatment Based on U-Net Predicted Masks and Original Masks Provided by a Human. All data are expressed in pixels. Table S3. Synthetic data for fixed orthodontics (T1) (tooth-based analysis, *n* = 44 each column). Table S4. Synthetic data for clear aligners (T2) (tooth-based analysis, *n* = 44 each column). Table S5. Synthetic data for fixed orthodontics (T1) (patient-based analysis, *n* = 11 each column). Table S6. Synthetic data for clear aligners (T2) (patient-based analysis, *n* = 11 each column).

## Abbreviations

The following abbreviations are used in this manuscript:

AI	Artificial Intelligence
CBCT	Cone-Beam Computed Tomography
DSC	Dice Similarity Coefficient
ERR	External Root Resorption
IoU	Intersection over Union
OPG	Orthopantomographic Radiograph
U-Net	Convolutional Neural Network Architecture for Image Segmentation
