# Supplementary material for: AI-Driven Quantitative Dental Imaging: A Clinical Framework for Assessing Root Resorption Across Treatment Modalities
Source: Dent J (Basel). 2026 Jun 25;14(7):392. doi: 10.3390/dj14070392 (PMC13409048; doi:10.3390/dj14070392)
Supplement: Supplementary file 1 [file dentistry-14-00392-s001.zip › dentistry-4314598-supplementary.pdf]

Table S1. Comparison of Tooth Length Measurements Before and After Fixed Orthodontic Treatment Based on U-Net Predicted Masks and Original Masks Provided by a Human. All data are expressed in pixels.

		**Fixed Orthodontics by AI**				**Fixed Orthodontics by Human**			
		Tooth 1	Tooth 2	Tooth 3	Tooth 4	Tooth 1	Tooth 2	Tooth 3	Tooth 4
Image 1	Before Length	160	161	150	159	160	131	159	159
	After Length	158	159	150	159	160	132	150	145
	Before -After	**2**	**2**	**0**	**0**	**0**	**-1**	**9**	**14**
Image 2	Before Length	152	148	145	160	160	138	123	120
	After Length	134	116	129	128	122	116	113	102
	Before -After	**18**	**32**	**16**	**32**	**38**	**22**	**10**	**18**
Image 3	Before Length	160	144	138	160	136	144	148	160
	After Length	160	132	131	126	135	142	142	128
	Before -After	**0**	**12**	**7**	**34**	**1**	**2**	**6**	**32**
Image 4	Before Length	134	137	137	158	136	151	153	135
	After Length	129	128	123	140	130	126	130	125
	Before -After	**5**	**11**	**14**	**18**	**6**	**25**	**23**	**10**
Image 5	Before Length	156	155	154	152	160	143	166	152
	After Length	144	156	117	142	158	140	140	141
	Before -After	**12**	**-1**	**37**	**10**	**2**	**3**	**26**	**11**
Image 6	Before Length	131	127	112	129	129	125	133	133
	After Length	110	104	110	105	127	117	115	130
	Before -After	**21**	**23**	**2**	**24**	**2**	**8**	**18**	**3**
Image 7	Before Length	129	130	129	132	143	118	128	157
	After Length	116	115	125	126	122	111	125	116
	Before -After	**13**	**115**	**4**	**6**	**21**	**7**	**3**	**41**
Image 8	Before Length	137	139	141	133	160	136	147	160
	After Length	123	135	126	131	137	135	131	141
	Before -After	**14**	**4**	**15**	**2**	**23**	**1**	**16**	**19**
Image 9	Before Length	133	128	128	132	126	128	126	126
	After Length	116	109	106	106	111	99	101	104
	Before -After	**17**	**19**	**22**	**26**	**15**	**29**	**25**	**22**
Image 10	Before Length	130	122	127	126	165	158	160	157
	After Length	128	122	118	119	161	136	135	155
	Before -After	**2**	**0**	**9**	**7**	**4**	**22**	**25**	**2**
Image 11	Before Length	134	126	120	129	145	132	126	138
	After Length	120	116	116	113	121	109	114	121
	Before -After	**14**	**10**	**4**	**16**	**24**	**23**	**12**	**17**

Table S2. Comparison of Tooth Length Measurements Before and After Clear Aligner Treatment Based on U-Net Predicted Masks and Original Masks Provided by a Human. All data are expressed in pixels.

		**Clear Aligner by AI**				**Clear Aligner by Human**			
		Tooth 1	Tooth 2	Tooth 3	Tooth 4	Tooth 1	Tooth 2	Tooth 3	Tooth 4
Image 1	Before Length	134	132	138	138	138	144	152	150
	After Length	122	132	135	134	130	139	135	137
	Before -After	**12**	**0**	**3**	**4**	**8**	**5**	**17**	**13**
Image 2	Before Length	133	133	135	134	140	126	129	140
	After Length	130	129	133	133	130	125	125	131
	Before -After	**3**	**4**	**2**	**1**	**10**	**1**	**4**	**9**
Image 3	Before Length	116	125	125	116	128	104	104	108
	After Length	112	110	117	118	106	87	90	102
	Before -After	**4**	**15**	**8**	**-2**	**22**	**17**	**14**	**6**
Image 4	Before Length	143	141	138	140	160	140	142	159
	After Length	140	137	132	139	160	135	141	159
	Before -After	**3**	**4**	**6**	**1**	**0**	**5**	**1**	**0**
Image 5	Before Length	141	143	139	138	140	145	150	149
	After Length	136	134	140	136	139	144	151	140
	Before -After	**5**	**9**	**-1**	**2**	**1**	**1**	**-1**	**9**
Image 6	Before Length	160	135	137	135	166	166	164	162
	After Length	161	126	136	131	162	165	158	161
	Before -After	**-1**	**9**	**1**	**4**	**4**	**1**	**6**	**1**
Image 7	Before Length	150	143	146	152	132	130	126	133
	After Length	148	137	138	143	117	122	124	124
	Before -After	**2**	**6**	**8**	**9**	**15**	**8**	**2**	**9**
Image 8	Before Length	160	159	160	163	160	160	160	160
	After Length	157	158	160	164	153	140	157	152
	Before -After	**3**	**1**	**0**	**-1**	**7**	**20**	**3**	**8**
Image 9	Before Length	160	150	150	148	160	158	158	158
	After Length	160	145	151	139	150	148	154	158
	Before -After	**0**	**5**	**-1**	**9**	**10**	**10**	**4**	**0**
Image 10	Before Length	160	160	158	161	160	160	160	160
	After Length	160	149	156	159	160	159	159	158
	Before -After	**0**	**11**	**2**	**2**	**0**	**1**	**1**	**2**
Image 11	Before Length	161	150	156	157	158	160	150	157
	After Length	152	139	142	158	136	147	134	142
	Before -After	**9**	**11**	**14**	**-1**	**22**	**13**	**16**	**15**

Table S3. Synthetic data for fixed orthodontics (T1) (**tooth-based analysis**, n=44 each column)

	T1 AI (i)	T1 AI (f)	T1 ΔAI	T1 Hum (i)	T1 Hum (f)	T1 ΔHum
Mean value	139.18	126.73	-12.45 (8.95%)	142.95	128.43	-14.52 (10.16%)
SD	13.20	15.57	10.06	14.36	15.85	10.87
Minimum	112	104	-37	118	99	-41
Maximum	161	160	1	166	161	1
Initial vs. final (p-value)	<0.0001**			<0.0001**		
Human vs AI (p-value)				0.1011	0.4759	0.381

**: highly significant

Table S4. Synthetic data for clear aligners (T2) (**tooth-based analysis**, n=44 each column)

	T2 AI (i)	T2 AI (f)	T2 ΔAI	T2 Hum (i)	T2 Hum (f)	T2 ΔHum
Mean value	142.20	138.20	-4.00 (2.81%)	144.80	137.59	-7.20 (4.98%)
SD	14.13	16.23	5.22	17.33	19.04	6.63
Min	112	103	-20	104	87	-22
Max	163	164	7	166	165	2
Initial vs. final (p-value)	<0.0001**			<0.0001**		
Human vs AI (p-value)				0.3642	0.6593	0.0133*
Fixed vs clear aligners			<0.0001**			0.001**

*: significant; **: highly significant

Table S5. Synthetic data for fixed orthodontics (T1) (**patient-based analysis**, n=11 each column)

	T1 AI (i)	T1 AI (f)	T1 ΔAI	T1 Hum (i)	T1 Hum (f)	T1 ΔHum
Mean value	139.18	126.73	-12.45	142.95	128.43	-14.52
SD	12.36	14.28	6.84	10.98	14.80	5.67
Minimum	124.75	107.25	-24.50	126.50	103.75	-22.75
Maximum	157.50	156.50	-1.00	160.00	146.75	-5.50
Initial vs. final (p-value)	0.001**			0.001**		
Human vs AI (p-value)				0.3737	0.9219	0.2662

*: significant

Table S6. Synthetic data for clear aligners (T2) (**patient-based analysis**, n=11 each column)

	T2 AI (i)	T2 AI (f)	T2 ΔAI	T2 Hum (i)	T2 Hum (f)	T2 ΔHum
Mean value	142.20	138.20	-4.00	144.80	137.59	-7.20
SD	13.50	15.12	2.24	17.27	19.13	5.35
Minimum	120.50	114.00	-8.25	111.00	96.25	-16.50
Maximum	160.50	159.75	-0.75	164.50	161.50	-0.75
Initial vs. final (p-value)	0.0038**			0.0233*		
Human vs AI (p-value)				0.6953	1.00	0.0618
Fixed vs clear aligners			0.0025**			0.0137*

*: significant; **: highly significant
